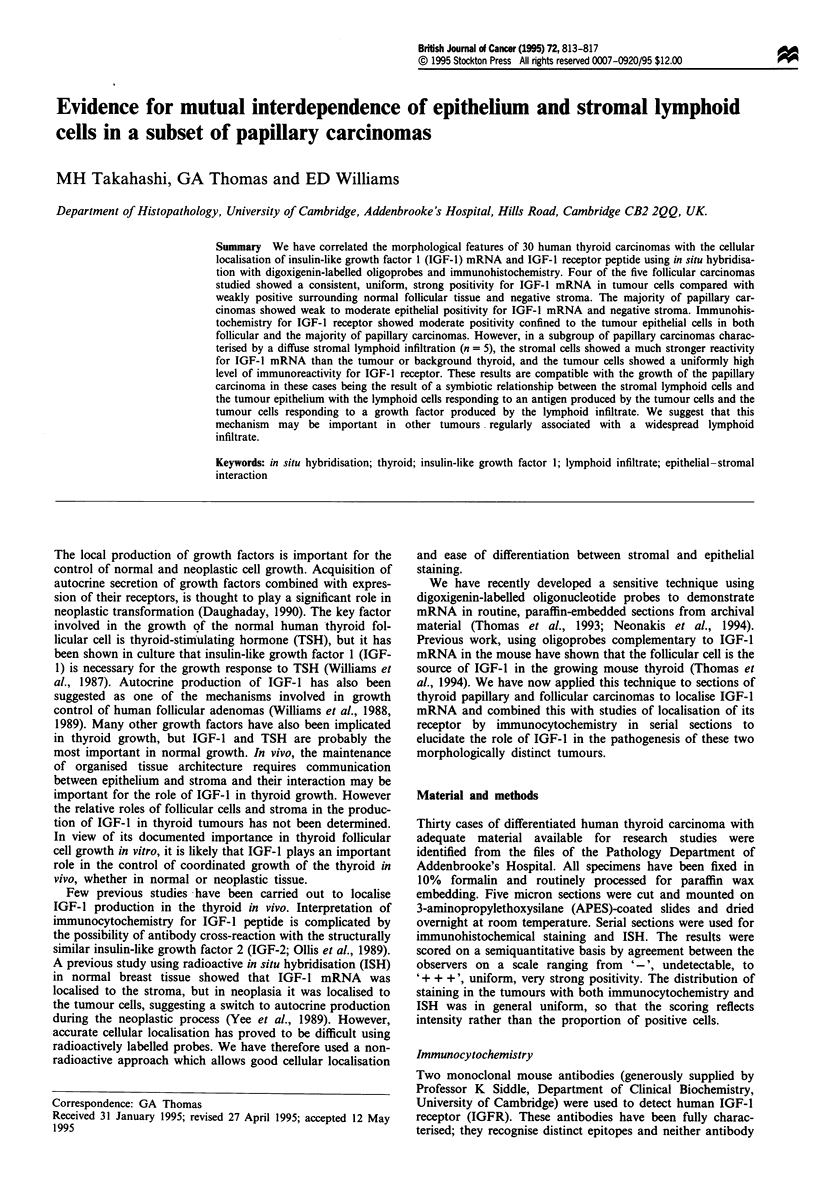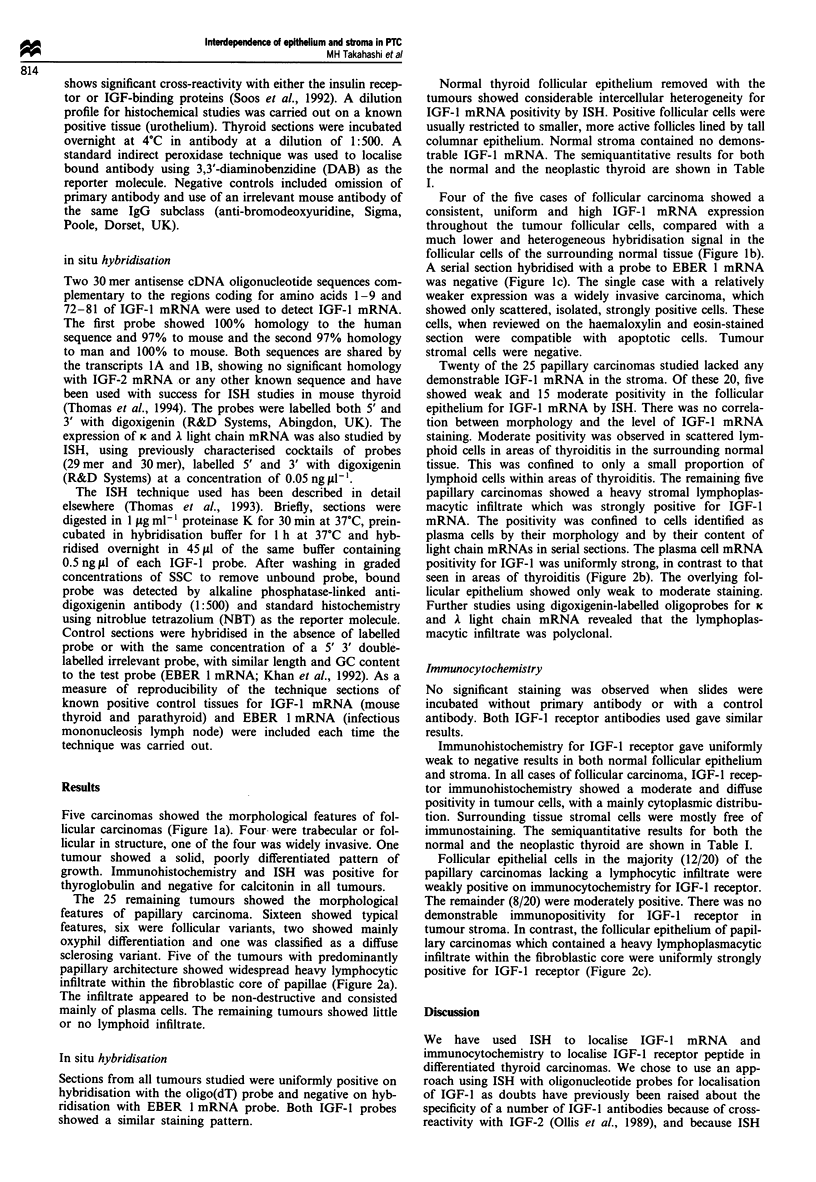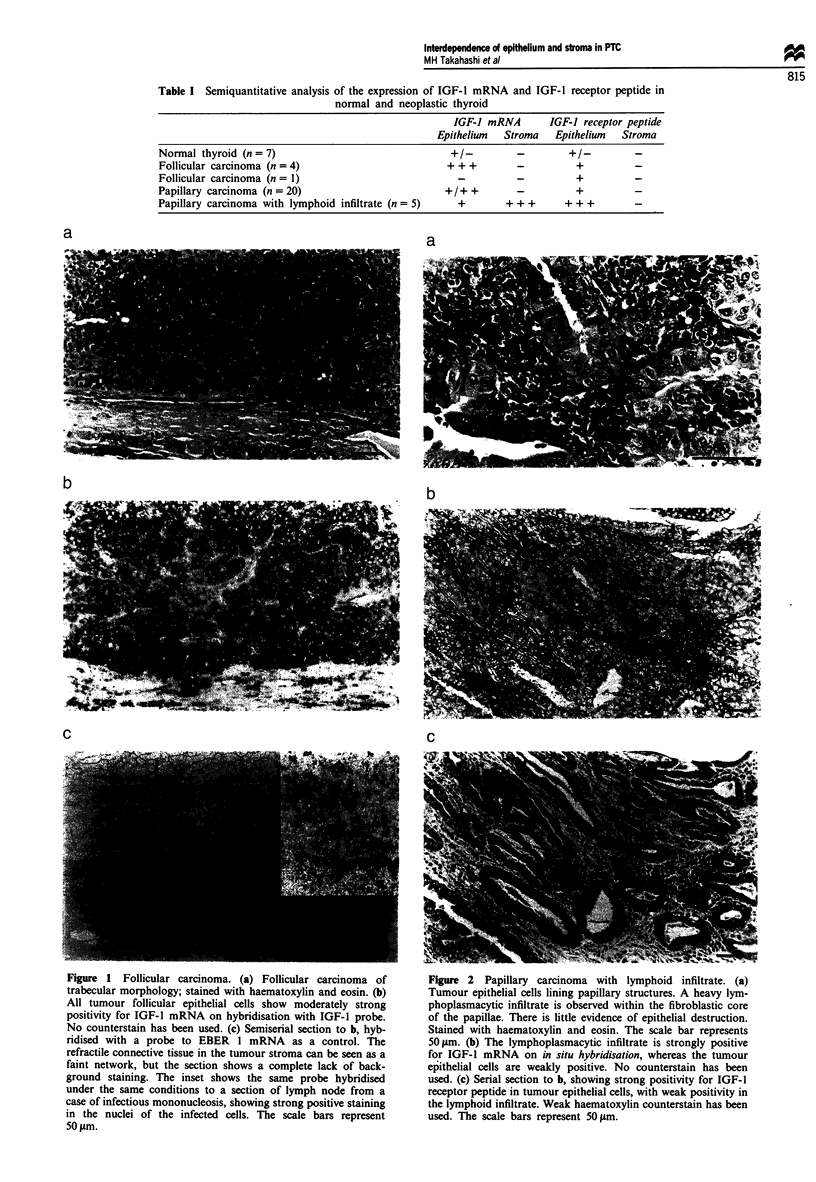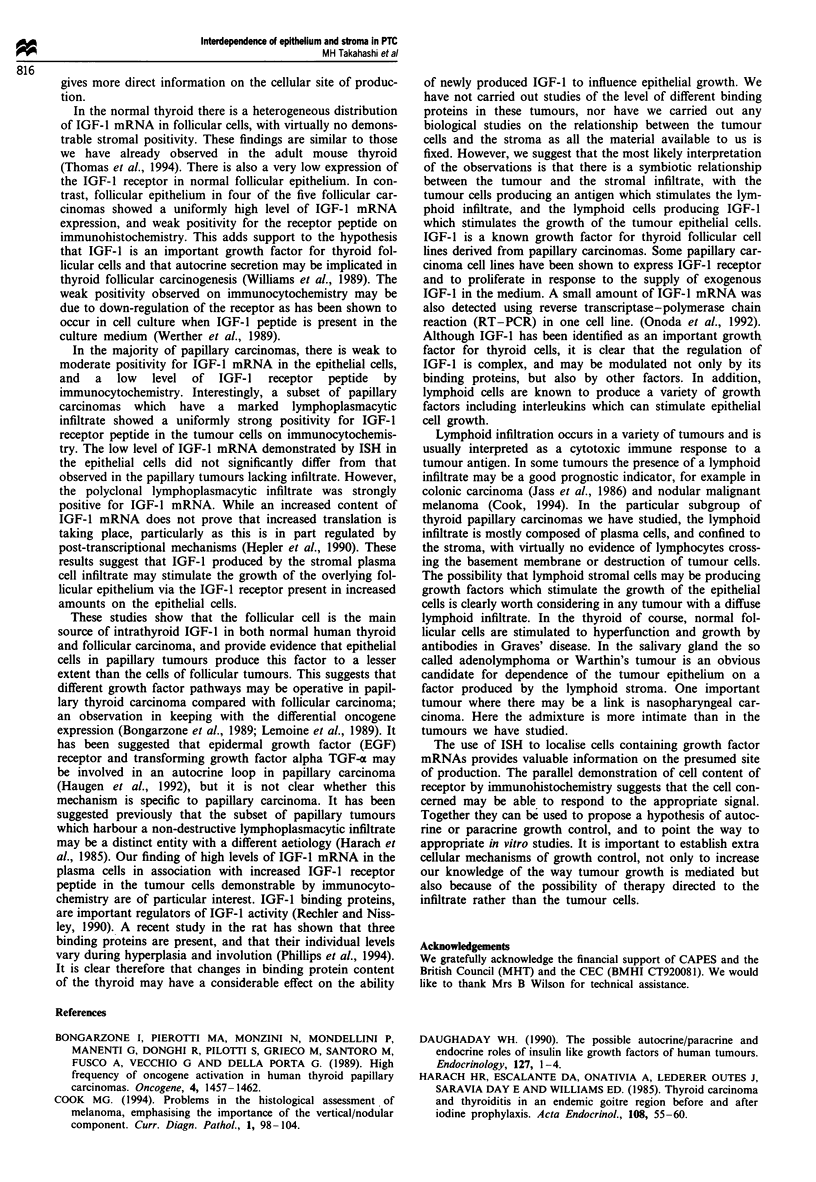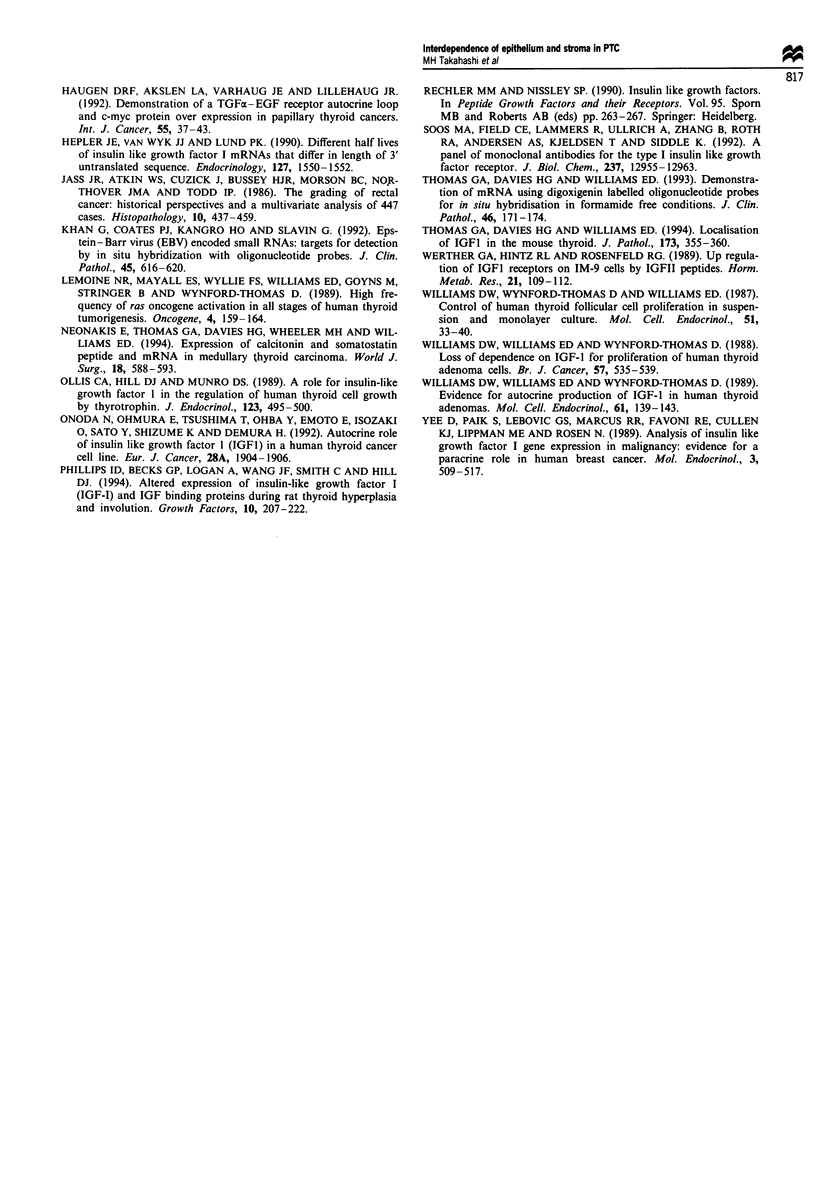# Evidence for mutual interdependence of epithelium and stromal lymphoid cells in a subset of papillary carcinomas.

**DOI:** 10.1038/bjc.1995.418

**Published:** 1995-10

**Authors:** M. H. Takahashi, G. A. Thomas, E. D. Williams

**Affiliations:** Department of Histopathology, University of Cambridge, Addenbrooke's Hospital, UK.

## Abstract

**Images:**


British Journal of Cancer (1995) 72, 813-817
© 1995 Stockton Press All rights reserved 0007-0920/95 $12.00

### Evidence for mutual interdependence of epithelium and stromal lymphoid cells in a subset of papillary carcinomas

MH Takahashi, GA Thomas and ED Williams

*Department of Histopathology, University of Cambridge, Addenbrooke's Hospital, Hills Road, Cambridge CB2 2QQ, UK.*

**Summary** We have correlated the morphological features of 30 human thyroid carcinomas with the cellular localisation of insulin-like growth factor 1 (IGF-1) mRNA and IGF-1 receptor peptide using *in situ* hybridisation with digoxigenin-labelled oligoprobes and immunohistochemistry. Four of the five follicular carcinomas studied showed a consistent, uniform, strong positivity for IGF-1 mRNA in tumour cells compared with weakly positive surrounding normal follicular tissue and negative stroma. The majority of papillary carcinomas showed weak to moderate epithelial positivity for IGF-1 mRNA and negative stroma. Immunohistochemistry for IGF-1 receptor showed moderate positivity confined to the tumour epithelial cells in both follicular and the majority of papillary carcinomas. However, in a subgroup of papillary carcinomas characterised by a diffuse stromal lymphoid infiltration ($n = 5$), the stromal cells showed a much stronger reactivity for IGF-1 mRNA than the tumour or background thyroid, and the tumour cells showed a uniformly high level of immunoreactivity for IGF-1 receptor. These results are compatible with the growth of the papillary carcinoma in these cases being the result of a symbiotic relationship between the stromal lymphoid cells and the tumour epithelium with the lymphoid cells responding to an antigen produced by the tumour cells and the tumour cells responding to a growth factor produced by the lymphoid infiltrate. We suggest that this mechanism may be important in other tumours regularly associated with a widespread lymphoid infiltrate.

**Keywords:** *in situ* hybridisation; thyroid; insulin-like growth factor 1; lymphoid infiltrate; epithelial-stromal interaction

The local production of growth factors is important for the control of normal and neoplastic cell growth. Acquisition of autocrine secretion of growth factors combined with expression of their receptors, is thought to play a significant role in neoplastic transformation (Daughaday, 1990). The key factor involved in the growth of the normal human thyroid follicular cell is thyroid-stimulating hormone (TSH), but it has been shown in culture that insulin-like growth factor 1 (IGF-1) is necessary for the growth response to TSH (Williams *et al.*, 1987). Autocrine production of IGF-1 has also been suggested as one of the mechanisms involved in growth control of human follicular adenomas (Williams *et al.*, 1988, 1989). Many other growth factors have also been implicated in thyroid growth, but IGF-1 and TSH are probably the most important in normal growth. *In vivo*, the maintenance of organised tissue architecture requires communication between epithelium and stroma and their interaction may be important for the role of IGF-1 in thyroid growth. However the relative roles of follicular cells and stroma in the production of IGF-1 in thyroid tumours has not been determined. In view of its documented importance in thyroid follicular cell growth *in vitro*, it is likely that IGF-1 plays an important role in the control of coordinated growth of the thyroid *in vivo*, whether in normal or neoplastic tissue.

Few previous studies have been carried out to localise IGF-1 production in the thyroid *in vivo*. Interpretation of immunocytochemistry for IGF-1 peptide is complicated by the possibility of antibody cross-reaction with the structurally similar insulin-like growth factor 2 (IGF-2; Ollis *et al.*, 1989). A previous study using radioactive *in situ* hybridisation (ISH) in normal breast tissue showed that IGF-1 mRNA was localised to the stroma, but in neoplasia it was localised to the tumour cells, suggesting a switch to autocrine production during the neoplastic process (Yee *et al.*, 1989). However, accurate cellular localisation has proved to be difficult using radioactively labelled probes. We have therefore used a non-radioactive approach which allows good cellular localisation and ease of differentiation between stromal and epithelial staining.

We have recently developed a sensitive technique using digoxigenin-labelled oligonucleotide probes to demonstrate mRNA in routine, paraffin-embedded sections from archival material (Thomas *et al.*, 1993; Neonakis *et al.*, 1994). Previous work, using oligoprobes complementary to IGF-1 mRNA in the mouse have shown that the follicular cell is the source of IGF-1 in the growing mouse thyroid (Thomas *et al.*, 1994). We have now applied this technique to sections of thyroid papillary and follicular carcinomas to localise IGF-1 mRNA and combined this with studies of localisation of its receptor by immunocytochemistry in serial sections to elucidate the role of IGF-1 in the pathogenesis of these two morphologically distinct tumours.

### Material and methods

Thirty cases of differentiated human thyroid carcinoma with adequate material available for research studies were identified from the files of the Pathology Department of Addenbrooke's Hospital. All specimens have been fixed in 10% formalin and routinely processed for paraffin wax embedding. Five micron sections were cut and mounted on 3-aminopropylethoxysilane (APES)-coated slides and dried overnight at room temperature. Serial sections were used for immunohistochemical staining and ISH. The results were scored on a semiquantitative basis by agreement between the observers on a scale ranging from '−', undetectable, to '+++', uniform, very strong positivity. The distribution of staining in the tumours with both immunocytochemistry and ISH was in general uniform, so that the scoring reflects intensity rather than the proportion of positive cells.

### *Immunocytochemistry*

Two monoclonal mouse antibodies (generously supplied by Professor K Siddle, Department of Clinical Biochemistry, University of Cambridge) were used to detect human IGF-1 receptor (IGFR). These antibodies have been fully characterised; they recognise distinct epitopes and neither antibody

Correspondence: GA Thomas
Received 31 January 1995; revised 27 April 1995; accepted 12 May 1995

shows significant cross-reactivity with either the insulin receptor or IGF-binding proteins (Soos *et al.*, 1992). A dilution profile for histochemical studies was carried out on a known positive tissue (urothelium). Thyroid sections were incubated overnight at 4°C in antibody at a dilution of 1:500. A standard indirect peroxidase technique was used to localise bound antibody using 3,3'-diaminobenzidine (DAB) as the reporter molecule. Negative controls included omission of primary antibody and use of an irrelevant mouse antibody of the same IgG subclass (anti-bromodeoxyuridine, Sigma, Poole, Dorset, UK).

### in situ *hybridisation*

Two 30 mer antisense cDNA oligonucleotide sequences complementary to the regions coding for amino acids 1–9 and 72–81 of IGF-1 mRNA were used to detect IGF-1 mRNA. The first probe showed 100% homology to the human sequence and 97% to mouse and the second 97% homology to man and 100% to mouse. Both sequences are shared by the transcripts 1A and 1B, showing no significant homology with IGF-2 mRNA or any other known sequence and have been used with success for ISH studies in mouse thyroid (Thomas *et al.*, 1994). The probes were labelled both 5' and 3' with digoxigenin (R&D Systems, Abingdon, UK). The expression of κ and λ light chain mRNA was also studied by ISH, using previously characterised cocktails of probes (29 mer and 30 mer), labelled 5' and 3' with digoxigenin (R&D Systems) at a concentration of 0.05 ng $\mu l^{-1}$.

The ISH technique used has been described in detail elsewhere (Thomas *et al.*, 1993). Briefly, sections were digested in 1 $\mu g\, ml^{-1}$ proteinase K for 30 min at 37°C, preincubated in hybridisation buffer for 1 h at 37°C and hybridised overnight in 45 μl of the same buffer containing 0.5 ng μl of each IGF-1 probe. After washing in graded concentrations of SSC to remove unbound probe, bound probe was detected by alkaline phosphatase-linked anti-digoxigenin antibody (1:500) and standard histochemistry using nitroblue tetrazolium (NBT) as the reporter molecule. Control sections were hybridised in the absence of labelled probe or with the same concentration of a 5' 3' double-labelled irrelevant probe, with similar length and GC content to the test probe (EBER 1 mRNA; Khan *et al.*, 1992). As a measure of reproducibility of the technique sections of known positive control tissues for IGF-1 mRNA (mouse thyroid and parathyroid) and EBER 1 mRNA (infectious mononucleosis lymph node) were included each time the technique was carried out.

### Results

Five carcinomas showed the morphological features of follicular carcinomas (Figure 1a). Four were trabecular or follicular in structure, one of the four was widely invasive. One tumour showed a solid, poorly differentiated pattern of growth. Immunohistochemistry and ISH was positive for thyroglobulin and negative for calcitonin in all tumours.

The 25 remaining tumours showed the morphological features of papillary carcinoma. Sixteen showed typical features, six were follicular variants, two showed mainly oxyphil differentiation and one was classified as a diffuse sclerosing variant. Five of the tumours with predominantly papillary architecture showed widespread heavy lymphocytic infiltrate within the fibroblastic core of papillae (Figure 2a). The infiltrate appeared to be non-destructive and consisted mainly of plasma cells. The remaining tumours showed little or no lymphoid infiltrate.

### In situ *hybridisation*

Sections from all tumours studied were uniformly positive on hybridisation with the oligo(dT) probe and negative on hybridisation with EBER 1 mRNA probe. Both IGF-1 probes showed a similar staining pattern.

Normal thyroid follicular epithelium removed with the tumours showed considerable intercellular heterogeneity for IGF-1 mRNA positivity by ISH. Positive follicular cells were usually restricted to smaller, more active follicles lined by tall columnar epithelium. Normal stroma contained no demonstrable IGF-1 mRNA. The semiquantitative results for both the normal and the neoplastic thyroid are shown in Table I.

Four of the five cases of follicular carcinoma showed a consistent, uniform and high IGF-1 mRNA expression throughout the tumour follicular cells, compared with a much lower and heterogeneous hybridisation signal in the follicular cells of the surrounding normal tissue (Figure 1b). A serial section hybridised with a probe to EBER 1 mRNA was negative (Figure 1c). The single case with a relatively weaker expression was a widely invasive carcinoma, which showed only scattered, isolated, strongly positive cells. These cells, when reviewed on the haemaloxylin and eosin-stained section were compatible with apoptotic cells. Tumour stromal cells were negative.

Twenty of the 25 papillary carcinomas studied lacked any demonstrable IGF-1 mRNA in the stroma. Of these 20, five showed weak and 15 moderate positivity in the follicular epithelium for IGF-1 mRNA by ISH. There was no correlation between morphology and the level of IGF-1 mRNA staining. Moderate positivity was observed in scattered lymphoid cells in areas of thyroiditis in the surrounding normal tissue. This was confined to only a small proportion of lymphoid cells within areas of thyroiditis. The remaining five papillary carcinomas showed a heavy stromal lymphoplasmacytic infiltrate which was strongly positive for IGF-1 mRNA. The positivity was confined to cells identified as plasma cells by their morphology and by their content of light chain mRNAs in serial sections. The plasma cell mRNA positivity for IGF-1 was uniformly strong, in contrast to that seen in areas of thyroiditis (Figure 2b). The overlying follicular epithelium showed only weak to moderate staining. Further studies using digoxigenin-labelled oligoprobes for κ and λ light chain mRNA revealed that the lymphoplasmacytic infiltrate was polyclonal.

### *Immunocytochemistry*

No significant staining was observed when slides were incubated without primary antibody or with a control antibody. Both IGF-1 receptor antibodies used gave similar results.

Immunohistochemistry for IGF-1 receptor gave uniformly weak to negative results in both normal follicular epithelium and stroma. In all cases of follicular carcinoma, IGF-1 receptor immunohistochemistry showed a moderate and diffuse positivity in tumour cells, with a mainly cytoplasmic distribution. Surrounding tissue stromal cells were mostly free of immunostaining. The semiquantitative results for both the normal and the neoplastic thyroid are shown in Table I.

Follicular epithelial cells in the majority (12/20) of the papillary carcinomas lacking a lymphocytic infiltrate were weakly positive on immunocytochemistry for IGF-1 receptor. The remainder (8/20) were moderately positive. There was no demonstrable immunopositivity for IGF-1 receptor in tumour stroma. In contrast, the follicular epithelium of papillary carcinomas which contained a heavy lymphoplasmacytic infiltrate within the fibroblastic core were uniformly strongly positive for IGF-1 receptor (Figure 2c).

### Discussion

We have used ISH to localise IGF-1 mRNA and immunocytochemistry to localise IGF-1 receptor peptide in differentiated thyroid carcinomas. We chose to use an approach using ISH with oligonucleotide probes for localisation of IGF-1 as doubts have previously been raised about the specificity of a number of IGF-1 antibodies because of cross-reactivity with IGF-2 (Ollis *et al.*, 1989), and because ISH

**Table I** Semiquantitative analysis of the expression of IGF-1 mRNA and IGF-1 receptor peptide in normal and neoplastic thyroid

| | *IGF-1 mRNA* | | *IGF-1 receptor peptide* | |
|---|---|---|---|---|
| | *Epithelium* | *Stroma* | *Epithelium* | *Stroma* |
| Normal thyroid ($n = 7$) | +/− | − | +/− | − |
| Follicular carcinoma ($n = 4$) | + + + | − | + | − |
| Follicular carcinoma ($n = 1$) | − | − | + | − |
| Papillary carcinoma ($n = 20$) | +/+ + | − | + | − |
| Papillary carcinoma with lymphoid infiltrate ($n = 5$) | + | + + + | + + + | − |

**Figure 1** Follicular carcinoma. (**a**) Follicular carcinoma of trabecular morphology; stained with haematoxylin and eosin. (**b**) All tumour follicular epithelial cells show moderately strong positivity for IGF-1 mRNA on hybridisation with IGF-1 probe. No counterstain has been used. (**c**) Semiserial section to **b**, hybridised with a probe to EBER 1 mRNA as a control. The refractile connective tissue in the tumour stroma can be seen as a faint network, but the section shows a complete lack of background staining. The inset shows the same probe hybridised under the same conditions to a section of lymph node from a case of infectious mononucleosis, showing strong positive staining in the nuclei of the infected cells. The scale bars represent 50 μm.

**Figure 2** Papillary carcinoma with lymphoid infiltrate. (**a**) Tumour epithelial cells lining papillary structures. A heavy lymphoplasmacytic infiltrate is observed within the fibroblastic core of the papillae. There is little evidence of epithelial destruction. Stained with haematoxylin and eosin. The scale bar represents 50 μm. (**b**) The lymphoplasmacytic infiltrate is strongly positive for IGF-1 mRNA on *in situ hybridisation*, whereas the tumour epithelial cells are weakly positive. No counterstain has been used. (**c**) Serial section to **b**, showing strong positivity for IGF-1 receptor peptide in tumour epithelial cells, with weak positivity in the lymphoid infiltrate. Weak haematoxylin counterstain has been used. The scale bars represent 50 μm.

gives more direct information on the cellular site of production.

In the normal thyroid there is a heterogeneous distribution of IGF-1 mRNA in follicular cells, with virtually no demonstrable stromal positivity. These findings are similar to those we have already observed in the adult mouse thyroid (Thomas *et al.*, 1994). There is also a very low expression of the IGF-1 receptor in normal follicular epithelium. In contrast, follicular epithelium in four of the five follicular carcinomas showed a uniformly high level of IGF-1 mRNA expression, and weak positivity for the receptor peptide on immunohistochemistry. This adds support to the hypothesis that IGF-1 is an important growth factor for thyroid follicular cells and that autocrine secretion may be implicated in thyroid follicular carcinogenesis (Williams *et al.*, 1989). The weak positivity observed on immunocytochemistry may be due to down-regulation of the receptor as has been shown to occur in cell culture when IGF-1 peptide is present in the culture medium (Werther *et al.*, 1989).

In the majority of papillary carcinomas, there is weak to moderate positivity for IGF-1 mRNA in the epithelial cells, and a low level of IGF-1 receptor peptide by immunocytochemistry. Interestingly, a subset of papillary carcinomas which have a marked lymphoplasmacytic infiltrate showed a uniformly strong positivity for IGF-1 receptor peptide in the tumour cells on immunocytochemistry. The low level of IGF-1 mRNA demonstrated by ISH in the epithelial cells did not significantly differ from that observed in the papillary tumours lacking infiltrate. However, the polyclonal lymphoplasmacytic infiltrate was strongly positive for IGF-1 mRNA. While an increased content of IGF-1 mRNA does not prove that increased translation is taking place, particularly as this is in part regulated by post-transcriptional mechanisms (Hepler *et al.*, 1990). These results suggest that IGF-1 produced by the stromal plasma cell infiltrate may stimulate the growth of the overlying follicular epithelium via the IGF-1 receptor present in increased amounts on the epithelial cells.

These studies show that the follicular cell is the main source of intrathyroid IGF-1 in both normal human thyroid and follicular carcinoma, and provide evidence that epithelial cells in papillary tumours produce this factor to a lesser extent than the cells of follicular tumours. This suggests that different growth factor pathways may be operative in papillary thyroid carcinoma compared with follicular carcinoma; an observation in keeping with the differential oncogene expression (Bongarzone *et al.*, 1989; Lemoine *et al.*, 1989). It has been suggested that epidermal growth factor (EGF) receptor and transforming growth factor alpha TGF-$\alpha$ may be involved in an autocrine loop in papillary carcinoma (Haugen *et al.*, 1992), but it is not clear whether this mechanism is specific to papillary carcinoma. It has been suggested previously that the subset of papillary tumours which harbour a non-destructive lymphoplasmacytic infiltrate may be a distinct entity with a different aetiology (Harach *et al.*, 1985). Our finding of high levels of IGF-1 mRNA in the plasma cells in association with increased IGF-1 receptor peptide in the tumour cells demonstrable by immunocytochemistry are of particular interest. IGF-1 binding proteins, are important regulators of IGF-1 activity (Rechler and Nissley, 1990). A recent study in the rat has shown that three binding proteins are present, and that their individual levels vary during hyperplasia and involution (Phillips *et al.*, 1994). It is clear therefore that changes in binding protein content of the thyroid may have a considerable effect on the ability of newly produced IGF-1 to influence epithelial growth. We have not carried out studies of the level of different binding proteins in these tumours, nor have we carried out any biological studies on the relationship between the tumour cells and the stroma as all the material available to us is fixed. However, we suggest that the most likely interpretation of the observations is that there is a symbiotic relationship between the tumour and the stromal infiltrate, with the tumour cells producing an antigen which stimulates the lymphoid infiltrate, and the lymphoid cells producing IGF-1 which stimulates the growth of the tumour epithelial cells. IGF-1 is a known growth factor for thyroid follicular cell lines derived from papillary carcinomas. Some papillary carcinoma cell lines have been shown to express IGF-1 receptor and to proliferate in response to the supply of exogenous IGF-1 in the medium. A small amount of IGF-1 mRNA was also detected using reverse transcriptase–polymerase chain reaction (RT–PCR) in one cell line. (Onoda *et al.*, 1992). Although IGF-1 has been identified as an important growth factor for thyroid cells, it is clear that the regulation of IGF-1 is complex, and may be modulated not only by its binding proteins, but also by other factors. In addition, lymphoid cells are known to produce a variety of growth factors including interleukins which can stimulate epithelial cell growth.

Lymphoid infiltration occurs in a variety of tumours and is usually interpreted as a cytotoxic immune response to a tumour antigen. In some tumours the presence of a lymphoid infiltrate may be a good prognostic indicator, for example in colonic carcinoma (Jass *et al.*, 1986) and nodular malignant melanoma (Cook, 1994). In the particular subgroup of thyroid papillary carcinomas we have studied, the lymphoid infiltrate is mostly composed of plasma cells, and confined to the stroma, with virtually no evidence of lymphocytes crossing the basement membrane or destruction of tumour cells. The possibility that lymphoid stromal cells may be producing growth factors which stimulate the growth of the epithelial cells is clearly worth considering in any tumour with a diffuse lymphoid infiltrate. In the thyroid of course, normal follicular cells are stimulated to hyperfunction and growth by antibodies in Graves' disease. In the salivary gland the so called adenolymphoma or Warthin's tumour is an obvious candidate for dependence of the tumour epithelium on a factor produced by the lymphoid stroma. One important tumour where there may be a link is nasopharyngeal carcinoma. Here the admixture is more intimate than in the tumours we have studied.

The use of ISH to localise cells containing growth factor mRNAs provides valuable information on the presumed site of production. The parallel demonstration of cell content of receptor by immunohistochemistry suggests that the cell concerned may be able to respond to the appropriate signal. Together they can be used to propose a hypothesis of autocrine or paracrine growth control, and to point the way to appropriate *in vitro* studies. It is important to establish extra cellular mechanisms of growth control, not only to increase our knowledge of the way tumour growth is mediated but also because of the possibility of therapy directed to the infiltrate rather than the tumour cells.

**Acknowledgements**
We gratefully acknowledge the financial support of CAPES and the British Council (MHT) and the CEC (BMHI CT920081). We would like to thank Mrs B Wilson for technical assistance.

**References**

BONGARZONE I, PIEROTTI MA, MONZINI N, MONDELLINI P, MANENTI G, DONGHI R, PILOTTI S, GRIECO M, SANTORO M, FUSCO A, VECCHIO G AND DELLA PORTA G. (1989). High frequency of oncogene activation in human thyroid papillary carcinomas. *Oncogene*, **4**, 1457–1462.

COOK MG. (1994). Problems in the histological assessment of melanoma, emphasising the importance of the vertical/nodular component. *Curr. Diagn. Pathol.*, **1**, 98–104.

DAUGHADAY WH. (1990). The possible autocrine/paracrine and endocrine roles of insulin like growth factors of human tumours. *Endocrinology*, **127**, 1–4.

HARACH HR, ESCALANTE DA, ONATIVIA A, LEDERER OUTES J, SARAVIA DAY E AND WILLIAMS ED. (1985). Thyroid carcinoma and thyroiditis in an endemic goitre region before and after iodine prophylaxis. *Acta Endocrinol.*, **108**, 55–60.

HAUGEN DRF, AKSLEN LA, VARHAUG JE AND LILLEHAUG JR. (1992). Demonstration of a TGFα-EGF receptor autocrine loop and c-myc protein over expression in papillary thyroid cancers. *Int. J. Cancer*, **55**, 37-43.

HEPLER JE, VAN WYK JJ AND LUND PK. (1990). Different half lives of insulin like growth factor I mRNAs that differ in length of 3' untranslated sequence. *Endocrinology*, **127**, 1550-1552.

JASS JR, ATKIN WS, CUZICK J, BUSSEY HJR, MORSON BC, NORTHOVER JMA AND TODD IP. (1986). The grading of rectal cancer: historical perspectives and a multivariate analysis of 447 cases. *Histopathology*, **10**, 437-459.

KHAN G, COATES PJ, KANGRO HO AND SLAVIN G. (1992). Epstein-Barr virus (EBV) encoded small RNAs: targets for detection by in situ hybridization with oligonucleotide probes. *J. Clin. Pathol.*, **45**, 616-620.

LEMOINE NR, MAYALL ES, WYLLIE FS, WILLIAMS ED, GOYNS M, STRINGER B AND WYNFORD-THOMAS D. (1989). High frequency of *ras* oncogene activation in all stages of human thyroid tumorigenesis. *Oncogene*, **4**, 159-164.

NEONAKIS E, THOMAS GA, DAVIES HG, WHEELER MH AND WILLIAMS ED. (1994). Expression of calcitonin and somatostatin peptide and mRNA in medullary thyroid carcinoma. *World J. Surg.*, **18**, 588-593.

OLLIS CA, HILL DJ AND MUNRO DS. (1989). A role for insulin-like growth factor 1 in the regulation of human thyroid cell growth by thyrotrophin. *J. Endocrinol.*, **123**, 495-500.

ONODA N, OHMURA E, TSUSHIMA T, OHBA Y, EMOTO E, ISOZAKI O, SATO Y, SHIZUME K AND DEMURA H. (1992). Autocrine role of insulin like growth factor 1 (IGF1) in a human thyroid cancer cell line. *Eur. J. Cancer*, **28A**, 1904-1906.

PHILLIPS ID, BECKS GP, LOGAN A, WANG JF, SMITH C AND HILL DJ. (1994). Altered expression of insulin-like growth factor I (IGF-I) and IGF binding proteins during rat thyroid hyperplasia and involution. *Growth Factors*, **10**, 207-222.

RECHLER MM AND NISSLEY SP. (1990). Insulin like growth factors. In *Peptide Growth Factors and their Receptors*. Vol. 95. Sporn MB and Roberts AB (eds) pp. 263-267. Springer: Heidelberg.

SOOS MA, FIELD CE, LAMMERS R, ULLRICH A, ZHANG B, ROTH RA, ANDERSEN AS, KJELDSEN T AND SIDDLE K. (1992). A panel of monoclonal antibodies for the type I insulin like growth factor receptor. *J. Biol. Chem.*, **237**, 12955-12963.

THOMAS GA, DAVIES HG AND WILLIAMS ED. (1993). Demonstration of mRNA using digoxigenin labelled oligonucleotide probes for *in situ* hybridisation in formamide free conditions. *J. Clin. Pathol.*, **46**, 171-174.

THOMAS GA, DAVIES HG AND WILLIAMS ED. (1994). Localisation of IGF1 in the mouse thyroid. *J. Pathol.*, **173**, 355-360.

WERTHER GA, HINTZ RL AND ROSENFELD RG. (1989). Up regulation of IGF1 receptors on IM-9 cells by IGFII peptides. *Horm. Metab. Res.*, **21**, 109-112.

WILLIAMS DW, WYNFORD-THOMAS D AND WILLIAMS ED. (1987). Control of human thyroid follicular cell proliferation in suspension and monolayer culture. *Mol. Cell. Endocrinol.*, **51**, 33-40.

WILLIAMS DW, WILLIAMS ED AND WYNFORD-THOMAS D. (1988). Loss of dependence on IGF-1 for proliferation of human thyroid adenoma cells. *Br. J. Cancer*, **57**, 535-539.

WILLIAMS DW, WILLIAMS ED AND WYNFORD-THOMAS D. (1989). Evidence for autocrine production of IGF-1 in human thyroid adenomas. *Mol. Cell. Endocrinol.*, **61**, 139-143.

YEE D, PAIK S, LEBOVIC GS, MARCUS RR, FAVONI RE, CULLEN KJ, LIPPMAN ME AND ROSEN N. (1989). Analysis of insulin like growth factor I gene expression in malignancy: evidence for a paracrine role in human breast cancer. *Mol. Endocrinol.*, **3**, 509-517.